# What We Do and Do Not Know about Teaching Medical Image Interpretation

**DOI:** 10.3389/fpsyg.2017.00309

**Published:** 2017-03-03

**Authors:** Ellen M. Kok, Koos van Geel, Jeroen J. G. van Merriënboer, Simon G. F. Robben

**Affiliations:** ^1^Department of Educational Development and Research, School of Health Professions Education, Maastricht UniversityMaastricht, Netherlands; ^2^Department of Radiology, Maastricht University Medical CentreMaastricht, Netherlands

**Keywords:** medical image interpretation, education, instructional design, e-learning, curriculum design, diagnostic reasoning, cognitive schemas, study strategies

## Abstract

Educators in medical image interpretation have difficulty finding scientific evidence as to how they should design their instruction. We review and comment on 81 papers that investigated instructional design in medical image interpretation. We distinguish between studies that evaluated complete offline courses and curricula, studies that evaluated e-learning modules, and studies that evaluated specific educational interventions. Twenty-three percent of all studies evaluated the implementation of complete courses or curricula, and 44% of the studies evaluated the implementation of e-learning modules. We argue that these studies have encouraging results but provide little information for educators: too many differences exist between conditions to unambiguously attribute the learning effects to specific instructional techniques. Moreover, concepts are not uniformly defined and methodological weaknesses further limit the usefulness of evidence provided by these studies. Thirty-two percent of the studies evaluated a specific interventional technique. We discuss three theoretical frameworks that informed these studies: diagnostic reasoning, cognitive schemas and study strategies. Research on diagnostic reasoning suggests teaching students to start with non-analytic reasoning and subsequently applying analytic reasoning, but little is known on how to train non-analytic reasoning. Research on cognitive schemas investigated activities that help the development of appropriate cognitive schemas. Finally, research on study strategies supports the effectiveness of practice testing, but more study strategies could be applicable to learning medical image interpretation. Our commentary highlights the value of evaluating specific instructional techniques, but further evidence is required to optimally inform educators in medical image interpretation.

## Introduction

*How to teach* medical image interpretation? For an educator in radiology, dermatology, pathology or cardiology, this might be the question in mind. Since ‘evidence-based medicine’ is held in high regard by clinicians, medical educators might aim to search the literature for evidence on how to design their instruction. Instructional design is the science and practical field of creating educational experiences (Merrill et al., 1996). This can be as broad as a curriculum or as narrow as a lesson, or even a single instructive animation. Unfortunately, evidence regarding how to teach medical image interpretation is hard to come by. Research on instructional design in medical image interpretation suffers from being scattered all over the literature, from a lack of cross-references and a lack of theoretical background. A lot of commentaries are published (e.g., Fenderson, 2005; Gunderman and Ballenger, 2014), which might serve as an inspiration, but should not be considered ‘evidence.’ This makes it challenging for medical educators to find and apply the relevant literature to their educational practice. The aim of this paper is twofold. On the one hand we synthesize existing literature about instructional design in medical image interpretation. On the other hand we identify gaps in the literature and propose research inspired by psychological theories as a solution. While we used an extensive literature search to inform our argument, the aim of this paper is not to be systematic and exhaustive, but to provide a commentary that is informed by a literature search.

## Methods

We searched Web of Science for papers related to instructional design, using the keywords (teach^∗^ OR education OR instruct^∗^ OR curric^∗^) AND [(Radiology OR Radiography) OR (pathology AND image) OR dermatology OR (electrocardiogra^∗^ OR ECG)]. We focused on papers less than 15 years old (2001- July 2016) to include recent papers only. This search yielded 4785 papers. Titles were scanned by EMK for relevance and subsequently the abstracts were scanned by KvG. 120 papers were selected for complete reading. EMK and KvG each checked half of these papers against the inclusion criteria. If doubt arose regarding the inclusion, both readers read the paper and discrepancy was resolved through discussion. We included only papers that implemented an educational experience and measured the effects of this intervention (against a control condition and/or against a pretest). We do not go into a discussion about what ‘effects’ of education are and should be, but consider ‘effective’ to be ‘yielding a higher score on a test,’ or ‘being evaluated more positively.’ We excluded papers where medical image interpretation was not the outcome measure (e.g., procedural knowledge), or that treated medical images as tools for teaching something else (e.g., use of radiographs as an illustration in anatomy classes). Table 1 in the Supplementary Materials provides an overview of the 81 selected papers, and they are marked with an asterisk in the reference list. We identified two broad categories of studies: (1) evaluations of curricula and courses (we discuss offline courses and e-learning courses separately) and (2) evaluations of specific instructional techniques. Three theoretical frameworks that form the basis of specific instructional techniques arose from the review: diagnostic reasoning, cognitive schemas, and study strategies. The curricula and courses in the first category commonly implement the specific instructional techniques in the second category of studies. However, studies in the first category rarely discuss specific instructional techniques, and, critically, these instructional techniques are not separately tested. We argue below that only evaluations of specific instructional techniques provide information that educators can use to design their education. In this paper, we discuss respectively evaluations of offline curricula and courses, evaluations of e-learning courses and evaluations of specific instructional techniques. For the purpose of the argument, we discuss representative papers in the rest of the manuscript and refer the reader to the Supplementary Materials for a complete overview of the reviewed papers.

## Review

### Evaluation of Offline Curricula and Courses

Twenty-three percent of the reviewed studies evaluated a course or curriculum. These are often a combination of lectures, workshops and self-study. The outcome of these studies might seem straight-forward and reassuring: the score on the post-test is typically higher than the score on the pretest, and the ‘new’ curriculum is more effective and more positively evaluated than the ‘old’ curriculum. However, appraisal of these results is problematic, for a variety of reasons. Firstly, numerous differences exist between the ‘new’ curriculum and the ‘old’ curriculum (or other control conditions). This makes the outcome ambiguous: it is impossible to know what makes a new curriculum more effective than the old curriculum. Possibly, the instructional techniques used in the new curriculum are more effective (but, if so, which of the techniques?). But the difference might just as well be caused by other factors, such as enthusiasm of the staff or students for the new curriculum. In line with Norman (2003), we argue that the evaluation of complete courses yields trivial findings that provide no insights for educators, unless the course is carefully compared with another course that *only* differs on specific, well-defined aspects. If seemingly more specific techniques are compared, such as case-based learning or self-directed learning, the findings provide hardly more insights. The techniques still differ in too many aspects, and often they are very broadly and not uniformly defined. This makes it even more difficult to compare the results over studies. On top of the problem that learning effects cannot be unambiguously attributed to specific instructional techniques, in many of the studies the methodology had apparent weaknesses, e.g., no control conditions and/or the use of inappropriate randomization.

### Evaluation of E-learning Modules

E-learning and blended learning are also widely investigated in medical image interpretation (44% of all papers). E-learning refers to learning activities that interactively use a computer to enhance learning (Ruiz et al., 2006). Blended learning refers to a mix of online with traditional (lecture-based) learning activities (Spanjers et al., 2015). E-learning and blended learning environments often provide participants with the opportunity to work through patient cases or provide content information in an interactive manner.

E-learning is a popular way to promote active learning: it allows for large groups of learners to engage in learning at a time and place convenient for them, has the potential to be tailored to learners’ needs, and allows for instructional designs that cannot be implemented in other formats (Cook et al., 2008). Certainly, e-learning is widely found to be more effective or non-inferior to traditional forms of teaching, in both medical education in general (Cook et al., 2008), and medical image interpretation (Zafar et al., 2014). Indeed, the lion’s share of the studies that we reviewed conform to this. Once again, the differences between the e-learning curriculum and the control condition (often a traditional, lecture-based curriculum) are often too large to unequivocally attribute effects to instructional techniques. This issue is even more pressing when it comes to e-learning. Not only do conditions differ from each other in terms of instructional techniques, the differences in the technology used for implementation further confound the comparison.

When an online or offline curriculum is implemented, it aims to maximize learning instead of isolating the contribution of specific educational techniques, even though a curriculum applies a set of instructional techniques. This means that studies resulting from this type of implementations are not optimized for providing specific information on how instruction should be designed. Indeed, many of the methodological weaknesses in this type of studies result from practical and ethical considerations (e.g., it is often impossible to randomly assign students to conditions). However, for an educator in medical image interpretation, it is important to know what specific techniques yield more effective learning, and therefore researchers need to design studies that answer this question. In the next section, we review studies that zoom in on specific instructional techniques. We argue that these specific studies are more informative for educators, not only because they provide more detailed information about ‘what works,’ but also because they are often theory-driven.

### Evaluation of Specific Instructional Techniques

Thirty-two percent of the studies that we reviewed evaluated a specific instructional technique. Most of these studies are (implicitly or explicitly) rooted in psychological theories. We discuss three psychological theories that together form the basis of most of these studies. We discuss respectively theories of diagnostic reasoning (Eva, 2004), cognitive schema theory (Charlin et al., 2007) and study strategies (Dunlosky et al., 2013).

#### Diagnostic Reasoning

Research in cognitive psychology proposes two modes of reasoning: analytic and non-analytic reasoning. Analytic reasoning refers to deliberate, effortful reasoning while non-analytic reasoning refers to automatic, rapid reasoning, also referred to as pattern recognition (Eva, 2004). Several studies use this framework to investigate specific educational techniques in medical image interpretation. For example, Ark et al. (2007) stimulated students to “carefully identify all features [of an ECG] while trusting guidance provided by feelings of familiarity,” i.e., balancing an analytical approach (carefully identifying features) and a non-analytical approach (trusting feelings of familiarity). This was more effective then not providing students with instructions on how to approach this task. Likewise, Baghdady et al. (2014a) found that students who were directed to diagnose a radiograph first and only then identify radiographic features outperformed participants who identified features first and then diagnosed the radiograph.

The claim that students should be instructed to diagnose a case first, based on feelings of familiarity (non-analytic reasoning) and only then collect and analyze all information (analytic reasoning) contrasts with the claim that it is crucial to systematically collect all relevant information in a medical image before making a diagnosis, which is the assumption underlying the idea of teaching a search pattern (Auffermann et al., 2015, 2016). While these studies provide evidence for a benefit of a search pattern training in radiology over no training, the benefit of systematic viewing training over a non-systematic search pattern training could not be established in radiology (Kok et al., 2016) or in ECG interpretation (Varvaroussis et al., 2014).

To sum up, these studies suggest teaching a balanced reasoning strategy, starting with non-analytic reasoning and subsequently applying analytic reasoning. Patel et al. (2015) suggest mapping and microanalysis as two tools to understand a learners’ reasoning process and provide focused feedback to train students in balancing reasoning strategies. Another option is to present students with high numbers of cases under time pressure, as a way to counteract the relatively high emphasis on analytical reasoning in medical education (Patel et al., 2015). These instructional techniques have not been investigated in medical image interpretation yet.

#### Cognitive Schemas

Diagnostic reasoning requires extensive knowledge that is structured into meaningful patterns, so-called cognitive schemas or illness scripts (Charlin et al., 2007). These contain information about pathophysiological processes underlying diseases, patients’ characteristics and signs and symptoms (Boshuizen and Schmidt, 1992; Van De Wiel et al., 2000). The acquisition of high-quality scripts is central to learning in medicine (Charlin et al., 2007) and thus several studies have explicitly or implicitly focused on helping students to develop high-quality scripts, often with a focus on either pathophysiological processes underlying diseases, patients characteristics or signs and symptoms.

Boshuizen and Schmidt (Boshuizen and Schmidt, 1992; Van De Wiel et al., 2000) argue that basic science knowledge (i.e., the understanding of pathophysiological processes underlying diseases) is fundamental to these illness scripts. However, with increased expertise, basic science knowledge is encapsulated and only used for difficult, atypical cases. Baghdady et al. (2009) argue that basic science knowledge helps diagnosis through creating a coherent mental representation of diseases and their (visual) features. Participants that were provided with causal explanations of radiological features learned more than students who were presented with feature lists or a structured algorithm. A second study, however, found that the negative effect of the structured algorithm (without basic science explanations) was mitigated by the previously discussed instruction to provide a diagnosis before summing up all features (Baghdady et al., 2014a).

The prevalence of a disease is included in illness scripts. Building solid information about actual prevalence of diseases into illness scripts can avoid the ‘prevalence bias’ in decision making (Croskerry, 2003). Pusic et al. (2012) found that the prevalence of normal and abnormal cases in a training set impacted the sensitivity-specificity trade-off, and should thus be considered when developing training sets.

Many studies have aimed to help students develop appropriate cognitive schemas that include the signs and symptoms of diseases. Blissett et al. (2015) suggest that expert-generated (well-structured) schemas can help students to understand the organization of knowledge. Indeed, they found improved learning (in the task of ECG interpretation) from expert-generated schemas as compared to learner-generated schemas. Dong et al. (2015) found that making concept maps (to explicitly structure knowledge) was more effective for learning ECG interpretation then traditional teaching. Other studies have found learning by comparison to be an effective way to teach signs and symptoms in radiology (Kok et al., 2013, 2015) and ECG interpretation (Ark et al., 2007). Medical image interpretation is also unique in that readers are often presented with two-dimensional representations of the three-dimensional body (van der Gijp et al., 2015). Two studies used 3D renderings and models to help participants understand the relationship between the signs and symptoms as seen in 2D and in 3D, in dermatology (Garg et al., 2010) and radiology (Lee et al., 2010).

Appropriate cognitive schemas are crucial for analytical and non-analytical reasoning. The development of appropriate cognitive schemas should thus be a key goal of instructional design. Further research into the optimal combination of these techniques, in order to connect pathophysiological processes underlying diseases, patients’ characteristics and signs and symptoms, is required. In particular, establishing a relationship between visual signs and symptoms, and verbal information about pathophysiological processes requires further research.

#### Study Strategies

Dunlosky et al. (2013) reviewed the effectiveness of 10 study strategies. Only two of those have been investigated in medical image interpretation: practice testing and mixed practice. Baghdady et al. (2014b) found practice testing to be a more effective way of studying dental radiology than engaging in additional study. Mixed practice (alternating practice on different kinds of problems) has been investigated by Hatala et al. (2003) and Shah et al. (2016). They compared blocked practice (practicing categories of abnormalities one by one) and mixed practice (practicing the items of the categories in mixed order). Shah et al. (2016) did not find differences in performance, while Hatala et al. (2003) did. While Dunlosky’s review considered the utility of the study strategies in diverse situations, many of those strategies are not applied to visual tasks, so the application of findings about effective study strategies in medical image interpretation requires further research.

## Conclusion

Instructional design in medical image interpretation has surpassed teacher-centered, lecture-based education and many examples of active, student-centered learning have successfully been implemented in medical image interpretation. However, the evaluation of those complete courses, curricula or e-learning modules provides few insights into specific techniques that lead to optimized learning. It is still unclear which techniques makes complete programs effective, and our educator is left with only a shallow understanding of what makes a specific instructional technique effective.

### Take-Home Messages for Educators

Our review of specific interventions provides more detailed recommendations, informed by theories about diagnostic reasoning, schema development and study strategies. It is suggested that educators should teach a balanced reasoning strategy, starting with non-analytic reasoning and subsequently applying analytic reasoning, although further research is required on how this should be taught. Building appropriate cognitive schemas is critical to teaching medical image interpretation, and several designs are proposed that support this. Concept-maps, learning through comparison and expert-generated schemas are found to be useful ways of supporting schema building. Finally, research on study strategies supports the effectiveness of practice testing, but more strategies could be applicable to medical image interpretation.

### Limitations

A limitation of this commentary is that we did not formally assess the quality of the reviewed studies. In general, randomized controlled trials are scarce and few studies included appropriate control conditions, although this problem was less prevalent in studies of specific interventions. A systematic assessment of study quality was beyond the scope of this literature-informed commentary but could be a relevant venue for further research. Furthermore, while this commentary discusses what conclusions cannot be drawn from the reviewed literature, there are many topics of which, as Rumsfeld said, ‘we do not know that we do not know them.’ Finally, we focused on outcome measures that reflect medical image interpretation. This excludes research on other important topics such as indications for imaging and professionalism.

Research on expertise in medical image interpretation states that experts have superior perceptual and cognitive abilities (Krupinski, 2010; Manning, 2010; Nodine and Mello-Thoms, 2010; Reingold and Sheridan, 2011; van der Gijp et al., 2014). Interestingly, few studies on education explicitly relate to research about visual expertise. Another remarkable gap in the literature is the lack of studies that focus on individualized and self-regulated learning, two important topics in instructional design nowadays (Van Merrienboer and Kirschner, 2013). In other visual domains, such as air traffic control (Salden et al., 2006), it was found that adapting training to the learners’ needs makes learning more efficient, so this finding is promising for medical image interpretation. Research on self-regulated learning in non-visual diagnostic reasoning provides a possible starting point for fostering self-regulated learning (Cleary et al., 2016). However, visual metacognition is found to be rather low (e.g., Võ et al., 2016), so it is important to understand how visual metacognition can be fostered in order to optimize learning medical image interpretation. In conclusion, we discussed findings relevant for teaching medical image interpretation, but many open questions remain, and further evidence is required to optimally inform educators in medical image interpretation.

## Author Contributions

The authors conceptualized the work together. EK and KvG conducted the review, EK wrote a first draft, all authors revised the work critically for important intellectual content. All authors approved the final version.

## Conflict of Interest Statement

The authors declare that the research was conducted in the absence of any commercial or financial relationships that could be construed as a potential conflict of interest.

The reviewer JS and the handling Editor declared their shared affiliation, and the handling Editor states that the process nevertheless met the standards of a fair and objective review.

## References

[B1] ^1^Al-Rawi W. T. Jacobs R. Hassan B. A. Sanderink G. Scarfe W. C. (2007). Evaluation of web-based instruction for anatomical interpretation in maxillofacial cone beam computed tomography. *Dentomaxillofac. Radiol.* 36 459–464. 10.1259/dmfr/25560514 18033941

[B2] ^1^Ark T. K. Brooks L. R. Eva K. W. (2006). Giving learners the best of both worlds: Do clinical teachers need to guard against teaching pattern recognition to novices?. *Acad. Med.* 81 405–409. 10.1097/00001888-200604000-00017 16565197

[B3] Ark T. K. Brooks L. R. Eva K. W. (2007). The benefits of flexibility: the pedagogical value of instructions to adopt multifaceted diagnostic reasoning strategies. *Med. Educ.* 41 281–287. 10.1111/j.1365-2929.2007.02688.x 17316213

[B4] ^1^Auffermann W. F. Henry T. S. Little B. P. Tigges S. Tridandapani S. (2015). Simulation for teaching and assessment of nodule perception on chest radiography in nonradiology health care trainees. *J. Am. Coll. Radiol.* 12 1215–1222. 10.1016/j.jacr.2015.07.014 26421854

[B5] ^1^Auffermann W. F. Little B. P. Tridandapani S. (2016). Teaching search patterns to medical trainees in an educational laboratory to improve perception of pulmonary nodules. *J. Med. Imaging* 3:011006 10.1117/1.JMI.3.1.011006 PMC4748144 26870749

[B6] ^1^Baghdady M. Carnahan H. Lam E. W. N. Woods N. N. (2014a). Dental and dental hygiene students’ diagnostic accuracy in oral radiology: effect of diagnostic strategy and instructional method. *J. Dent. Educ.* 78 1279–1285. 25179924

[B7] ^1^Baghdady M. Carnahan H. Lam E. W. N. Woods N. N. (2014b). Test-enhanced learning and its effect on comprehension and diagnostic accuracy. *Med. Educ.* 48 181–188. 10.1111/medu.12302 24528400

[B8] ^1^Baghdady M. Pharoah M. J. Regehr G. Lam E. W. N. Woods N. N. (2009). The role of basic sciences in diagnostic oral radiology. *J. Dent. Educ.* 73 1187–1193. 19805783

[B9] ^1^Bailey J. H. Roth T. D. Kohli M. D. Heitkamp D. E. (2014). Real view radiology – impact on search patterns and confidence in radiology education. *Acad. Radiol.* 21 859–868. 10.1016/j.acra.2013.11.022 24820675

[B10] ^1^Blissett S. Cavalcanti R. Sibbald M. (2015). ECG rhythm analysis with expert and learner-generated schemas in novice learners. *Adv. Health Sci. Educ.* 20 915–933. 10.1007/s10459-014-9572-y 25481281

[B11] ^1^Boespflug A. Guerra J. Dalle S. Thomas L. (2015). Enhancement of customary dermoscopy education with spaced education e-learning a prospective controlled trial. *JAMA Dermatol.* 151 847–853. 10.1001/jamadermatol.2015.0214 25902339

[B12] ^1^Bojsen S. R. Rader S. Holst A. G. Kayser L. Ringsted C. Svendsen J. H. (2015). The acquisition and retention of ECG interpretation skills after a standardized web-based ECG tutorial-a randomised study. *BMC Med. Educ.* 15:36 10.1186/s12909-015-0319-0 PMC4356122 25889642

[B13] Boshuizen H. P. Schmidt H. G. (1992). On the role of biomedical knowledge in clinical reasoning by experts, intermediates and novices. *Cogn. Sci.* 16 153–184. 10.1207/s15516709cog1602_1

[B14] ^1^Breen C. Zhu T. T. Bond R. Finlay D. Clifford G. (2016). The evaluation of an open source online training system for teaching 12 lead electrocardiographic interpretation. *J. Electrocardiol.* 49 454–461. 10.1016/j.jelectrocard.2016.02.003 26925494

[B15] ^1^Burbridge B. Kalra N. Malin G. Trinder K. Pinelle D. (2015). University of saskatchewan radiology courseware (USRC): an assessment of its utility for teaching diagnostic imaging in the medical school curriculum. *Teach. Learn. Med.* 27 91–98. 10.1080/10401334.2014.979180 25584477

[B16] ^1^Ceresnak S. R. Axelrod D. M. Motonaga K. S. Johnson E. R. Krawczeski C. D. (2016). Pediatric cardiology boot camp: description and evaluation of a novel intensive training program for pediatric cardiology trainees. *Pediatr. Cardiol.* 37 834–844. 10.1007/s00246-016-1357-z 26961569

[B17] Charlin B. Boshuizen H. Custers E. J. Feltovich P. J. (2007). Scripts and clinical reasoning. *Med. Educ.* 41 1178–1184. 10.1111/j.1365-2923.2007.02924.x 18045370

[B18] ^1^Chudakoff J. H. Obuchowski N. A. Mehta N. Reid J. R. (2013). Worldwide utilization of a web-based learning tool for pediatric radiology. *AJR Am. J. Roentgenol.* 200 974–979. 10.2214/ajr.12.10443 23617478

[B19] ^1^Chudgar S. M. Engle D. L. Grochowski C. O. Gagliardi J. P. (2016). Teaching crucial skills: an electrocardiogram teaching module for medical students. *J. Electrocardiol.* 49 490–495. 10.1016/j.jelectrocard.2016.03.021 27083329

[B20] Cleary T. J. Durning S. J. Artino A. R. Jr. (2016). Microanalytic assessment of self-regulated learning during clinical reasoning tasks: recent developments and next steps. *Acad. Med.* 91 1516–1521. 10.1097/ACM.0000000000001228 27191840

[B21] ^1^Cliff S. Bedlow A. J. Melia J. Moss S. Harland C. C. (2003). Impact of skin cancer education on medical students’ diagnostic skills. *Clin. Exp. Dermatol.* 28 214–217. 10.1046/j.1365-2230.2003.01237.x 12653717

[B22] Cook D. A. Levinson A. J. Garside S. Dupras D. M. Erwin P. J. Montori V. M. (2008). Internet-based learning in the health professions: a meta-analysis. *J. Am. Med. Assoc.* 300 1181–1196. 10.1001/jama.300.10.1181 18780847

[B23] Croskerry P. (2003). The importance of cognitive errors in diagnosis and strategies to minimize them. *Acad. Med.* 78 775–780. 10.1097/00001888-200308000-00003 12915363

[B24] ^1^Darras K. E. Worthington A. Russell D. Hou D. J. Forster B. B. Hague C. J. (2016). Implementation of a longitudinal introduction to radiology course during internship year improves diagnostic radiology residents’ academic and clinical skills: a Canadian experience. *Acad. Radiol.* 23 848–860. 10.1016/j.acra.2016.03.007 27178649

[B25] ^1^DeBonis K. Blair T. R. Payne S. T. Wigan K. Kim S. (2015). Viability of a web-based module for teaching electrocardiogram reading skills to psychiatry residents: learning outcomes and trainee interest. *Acad. Psychiatry* 39 645–648. 10.1007/s40596-014-0249-x 25391493

[B26] ^1^Dong R. M. Yang X. Y. Xing B. R. Zou Z. H. Zheng Z. D. Xie X. J. (2015). Use of concept maps to promote electrocardiogram diagnosis learning in undergraduate medical students. *Int. J. Clin. Exp. Med.* 8 7794–7801. 26221331 PMC4509276

[B27] Dunlosky J. Rawson K. A. Marsh E. J. Nathan M. J. Willingham D. T. (2013). Improving students’ learning with effective learning techniques promising directions from cognitive and educational psychology. *Psychol. Sci.* 14 4–58. 10.1177/1529100612453266 26173288

[B28] Eva K. W. (2004). What every teacher needs to know about clinical reasoning. *Med. Educ.* 39 98–106. 10.1111/j.1365-2929.2004.01972.x 15612906

[B29] ^1^Eva K. W. Hatala R. M. LeBlanc V. R. Brooks L. R. (2007). Teaching from the clinical reasoning literature: combined reasoning strategies help novice diagnosticians overcome misleading information. *Med. Educ.* 41 1152–1158. 10.1111/j.1365-2923.2007.02923.x 18045367

[B30] ^1^Fawcett R. S. Widmaier E. J. Cavanaugh S. H. (2004). Digital technology enhances dermatology teaching in a family medicine residency. *Fam. Med.* 36 89–91. 14872352

[B31] Fenderson B. A. (2005). Strategies for teaching pathology to graduate students and allied health professionals. *Hum. Pathol.* 36 146–153. 10.1016/j.humpath.2004.09.022 15754291

[B32] ^1^Garg A. Haley H. L. Hatem D. (2010). Modern moulage evaluating the use of 3-dimensional prosthetic mimics in a dermatology teaching program for second-year medical students. *Arch. Dermatol.* 146 143–146. 10.1001/archdermatol.2009.355 20157024

[B33] ^1^Giunta A. Di Stefani A. Chimenti S. (2011). Mobile phones: a role in teaching dermatology? *Dermatology* 222 22–23. 10.1159/000317074 20720386

[B34] Gunderman R. B. Ballenger Z. (2014). The golden rule of education. *Acad. Radiol.* 21 1078–1079. 10.1016/j.acra.2014.01.026 25018080

[B35] ^1^Hatala R. M. Brooks L. R. Norman G. R. (2003). Practice makes perfect: the critical role of mixed practice in the acquisition of ECG interpretation skills. *Adv. Health Sci. Educ.* 8 17–26. 10.1023/a:1022687404380 12652166

[B36] ^1^Hecht S. Adams W. H. Cunningham M. A. Lane I. F. Howell N. E. (2013). Student performance and course evaluations before and after use of the classroom performance system (tm) in a third-year veterinary radiology course. *Vet. Radiol. Ultrasound* 54 114–121. 10.1111/vru.12001 23240856

[B37] ^1^Jenkins S. Goel R. Morrell D. S. (2008). Computer-assisted instruction versus traditional lecture for medical student teaching of dermatology morphology: a randomized control trial. *J. Am. Acad. Dermatol.* 59 255–259. 10.1016/j.jaad.2008.04.026 18499299

[B38] ^1^Kaliyadan F. Amri M. Dhufiri M. Amin T. T. Khan M. A. (2012). Effectiveness of a modified tutorless problem-based learning method in dermatology - a pilot study. *J. Eur. Acad. Dermatol. Venereol.* 26 111–113. 10.1111/j.1468-3083.2011.04016.x 21366714

[B39] ^1^Kavadella A. Tsiklakis K. Vougiouklakis G. Lionarakis A. (2012). Evaluation of a blended learning course for teaching oral radiology to undergraduate dental students. *Eur. J. Dent. Educ.* 16 E88–E95. 10.1111/j.1600-0579.2011.00680.x 22251359

[B40] ^1^Ketelsen D. Schrodl F. Knickenberg I. Heckemann R. A. Hothorn T. Neuhuber W. L. (2007). Modes of information delivery in radiologic anatomy education: impact on student performance. *Acad. Radiol.* 14 93–99. 10.1016/j.acra.2006.10.013 17236274

[B41] ^1^Kohlwes R. J. Shank R. (2005). An electrocardiogram curriculum for resident doctors. *Med. Educ.* 39 1163–1164. 10.1111/j.1365-2929.2005.02284.x 16262838

[B42] ^1^Kok E. M. de Bruin A. B. H. Leppink J. van Merrienboer J. J. G. Robben S. G. F. (2015). Case comparisons: an efficient way of learning radiology. *Acad. Radiol.* 22 1226–1235. 10.1016/j.acra.2015.04.012 26254543

[B43] ^1^Kok E. M. de Bruin A. B. H. Robben S. C. F. van Merrienboer J. J. G. (2013). Learning radiological appearances of diseases: Does comparison help? *Learn. Instr.* 23 90–97. 10.1016/j.learninstruc.2012.07.004

[B44] ^1^Kok E. M. Jarodzka H. de Bruin A. B. H. BinAmir H. A. N. Robben S. G. F. van Merrienboer J. J. G. (2016). Systematic viewing in radiology: seeing more, missing less? *Adv. Health Sci. Educ.* 21 189–205. 10.1007/s10459-015-9624-y PMC4749649 26228704

[B45] Krupinski E. A. (2010). “Perceptual factors in reading medical images,” in *The Handbook of Medical Image Perception and Techniques*, eds Samei E. Krupinski E. (Cambridge: Cambridge University Press), 81–90.

[B46] ^1^Lavranos G. Koliaki C. Briasoulis A. Nikolaou A. Stefanadis C. (2013). Effectiveness of current teaching methods in Cardiology: the SKILLS (medical Students knowledge integration of lower level clinical skills) study. *Hippokratia* 17 34–37. 23935341 PMC3738274

[B47] ^1^Lee B. F. Chiu N. T. Li C. Y. (2013). Value of case-based learning in a nuclear medicine clerkship. *J. Am. Coll. Radiol.* 10 135–141. 10.1016/j.jacr.2012.07.015 23374691

[B48] ^1^Lee H. Kim J. Cho Y. Kim M. Kim N. Lee K. (2010). Three-dimensional computed tomographic volume rendering imaging as a teaching tool in veterinary radiology instruction. *Vet. Med.* 55 603–609.

[B49] ^1^Li J. Li Q. L. Li J. Chen M. L. Xie H. F. Li Y. P. (2013). Comparison of three problem-based learning conditions (real patients, digital and paper) with lecture-based learning in a dermatology course: a prospective randomized study from China. *Med. Teach.* 35 E963–E970. 10.3109/0142159x.2012.719651 23009254

[B50] ^1^Lieberman G. Abramson R. Volkan K. McArdle P. J. (2002). Tutor versus computer: a prospective comparison of interactive tutorial and computer-assisted instruction in radiology education. *Acad. Radiol.* 9 40–49. 10.1016/s1076-6332(03)80295-7 11918358

[B51] ^1^Liebman T. N. Goulart J. M. Soriano R. Dusza S. W. Halpern A. C. Lee K. K. (2012). Effect of dermoscopy education on the ability of medical students to detect skin cancer. *Arch. Dermatol.* 148 1016–1022. 10.1001/archdermatol.2012.509 22986850

[B52] ^1^Lim-Dunham J. E. Ensminger D. C. McNulty J. A. Hoyt A. E. Chandrasekhar A. J. (2016). A vertically integrated online radiology curriculum developed as a cognitive apprenticeship: impact on student performance and learning. *Acad. Radiol.* 23 252–261. 10.1016/j.acra.2015.09.018 26719161

[B53] ^1^Mahler S. A. Wolcott C. J. Swoboda T. K. Wang H. Arnold T. C. (2011). Techniques for teaching electrocardiogram interpretation: self-directed learning is less effective than a workshop or lecture. *Med. Educ.* 45 347–353. 10.1111/j.1365-2923.2010.03891.x 21401682

[B54] ^1^Mahnken A. H. Baumann M. Meister M. Schmitt V. Fischer M. R. (2011). Blended learning in radiology: Is self-determined learning really more effective? *Eur. J. Radiol.* 78 384–387. 10.1016/j.ejrad.2010.12.059 21288674

[B55] ^1^Maleck M. Fischer M. R. Kammer B. Zeiler C. Mangel E. Schenk F. (2001). Do computers teach better? A media comparison study for case-based teaching in radiology. *Radiographics* 21 1025–1032. 10.1148/radiographics.21.4.g01jl091025 11452078

[B56] ^1^Maloney E. Hippe D. S. Paladin A. Chew F. S. Ha A. S. (2016). Musculoskeletal ultrasound training for radiology residents: lecture versus interactive learning module. *Acad. Radiol.* 23 789–796. 10.1016/j.acra.2015.11.018 27067604

[B57] Manning D. J. (2010). “Cognitive factors in reading medical images,” in *The Handbook of Medical Image Perception and Techniques*, eds Samei E. Krupinski E. (Cambridge: Cambridge University Press), 91–106.

[B58] ^1^Marsch A. F. Espiritu B. Groth J. Hutchens K. A. (2014). The effectiveness of annotated (vs. non-annotated) digital pathology slides as a teaching tool during dermatology and pathology residencies. *J. Cutan. Pathol.* 41 513–518. 10.1111/cup.12328 24620842

[B59] ^1^Meckfessel S. Stuhmer C. Bormann K. H. Kupka T. Behrends M. Matthies H. (2011). Introduction of e-learning in dental radiology reveals significantly improved results in final examination. *J. Cranio Maxillofac. Surg.* 39 40–48. 10.1016/j.jcms.2010.03.008 20452231

[B60] Merrill M. D. Drake L. Lacy M. J. Pratt J. Group I. R. (1996). Reclaiming instructional design. *Educ. Technol.* 36 5–7.

[B61] ^1^Mileman P. A. van den Hout W. B. Sanderink G. C. H. (2003). Randomized controlled trial of a computer-assisted learning program to improve caries detection from bitewing radiographs. *Dentomaxillofac. Radiol.* 32 116–123. 10.1259/dmfr/58225203 12775666

[B62] ^1^Montassier E. Hardouin J. B. Segard J. Batard E. Potel G. Planchon B. (2016). e-Learning versus lecture-based courses in ECG interpretation for undergraduate medical students: a randomized noninferiority study. *Eur. J. Emerg. Med.* 23 108–113. 10.1097/mej.0000000000000215 25386694

[B63] ^1^Nilsson M. Bolinder G. Held C. Johansson B. L. Fors U. Ostergren J. (2008). Evaluation of a web-based ECG-interpretation programme for undergraduate medical students. *BMC Med. Educ.* 8:25 10.1186/1472-6920-8-25 PMC2394519 18430256

[B64] Nodine C. Mello-Thoms C. (2010). “The role of expertise in radiologic image interpretation,” in *The Handbook of Medical Image Perception and Techniques*, eds Samei E. Krupinski E. (Cambridge: Cambridge University Press), 139–156.

[B65] Norman G. (2003). RCT = results confounded and trivial: the perils of grand educational experiments. *Med. Educ.* 37 582–584. 10.1046/j.1365-2923.2003.01586.x 12834412

[B66] ^1^Ochsendorf F. R. Boehncke W. H. Boer A. Kaufmann R. (2004). Prospective randomised comparison of traditional, personal bedside and problem-oriented practical dermatology courses. *Med. Educ.* 38 652–658. 10.1111/j.1365-2929.2004.01838.x 15189262

[B67] ^1^Ochsendorf F. R. Boehncke W. H. Sommerlad M. Kaufmann R. (2006). Interactive large-group teaching in a dermatology course. *Med. Teach.* 28 697–701. 10.1080/01421590601034241 17594580

[B68] ^1^O’Connor E. E. Fried J. McNulty N. Shah P. Hogg J. P. Lewis P. (2016). Flipping radiology education right side up. *Acad. Radiol.* 23 810–822. 10.1016/j.acra.2016.02.011 27066755

[B69] ^1^Pagnanelli G. Soyer H. P. Argenziano G. Talamini R. Barbati R. Bianchi L. (2003). Diagnosis of pigmented skin lesions by dermoscopy: web-based training improves diagnostic performance of non-experts. *Br. J. Dermatol.* 148 698–702. 10.1046/j.1365-2133.2003.05168.x 12752126

[B70] Patel R. Sandars J. Carr S. (2015). Clinical diagnostic decision-making in real life contexts: a trans-theoretical approach for teaching: AMEE Guide No. 95. *Med. Teach.* 37 211–227. 10.3109/0142159X.2014.975195 25391895

[B71] ^1^Porras L. Drezner J. Dotson A. Stafford H. Berkoff D. Agnihotri K. (2016). Novice interpretation of screening electrocardiograms and impact of online training. *J. Electrocardiol.* 49 462–466. 10.1016/j.jelectrocard.2016.02.004 27055937

[B72] ^1^Porter K. K. Bailey P. D. Woods R. Scott W. W. Johnson P. T. (2015). Retained surgical item identification on imaging studies: a training module for radiology residents. *Int. J. Comput. Assist. Radiol. Surg.* 10 1803–1809. 10.1007/s11548-015-1154-9 25673074

[B73] ^1^Pusic M. V. Andrews J. S. Kessler D. O. Teng D. C. Pecaric M. R. Ruzal-Shapiro C. (2012). Prevalence of abnormal cases in an image bank affects the learning of radiograph interpretation. *Med. Educ.* 46 289–298. 10.1111/j.1365-2923.2011.04165.x 22324528

[B74] ^1^Pusic M. V. LeBlanc V. R. Miller S. Z. (2007). Linear versus web-style layout of computer tutorials for medical student learning of radiograph interpretation. *Acad. Radiol.* 14 877–889. 10.1016/j.acra.2007.04.013 17574137

[B75] ^1^Raupach T. Brown J. Anders S. Hasenfuss G. Harendza S. (2013). Summative assessments are more powerful drivers of student learning than resource intensive teaching formats. *BMC Med.* 11:10 10.1186/1741-7015-11-61 PMC3635879 23497243

[B76] ^1^Raupach T. Hanneforth N. Anders S. Pukrop T. ten Cate O. T. J. Harendza S. (2010). Impact of teaching and assessment format on electrocardiogram interpretation skills. *Med. Educ.* 44 731–740. 10.1111/j.1365-2923.2010.03687.x 20528994

[B77] ^1^Raupach T. Harendza S. Anders S. Schuelper N. Brown J. (2016). How can we improve teaching of ECG interpretation skills? Findings from a prospective randomised trial. *J. Electrocardiol.* 49 7–12. 10.1016/j.jelectrocard.2015.10.004 26615874

[B78] Reingold E. M. Sheridan H. (2011). “Eye movements and visual expertise in chess and medicine,” in *The Oxford Handbook of Eye Movements*, eds Leversedge S. P. Gilchrist I. D. Everling S. (Oxford: Oxford University Press), 528–550. 10.1093/oxfordhb/9780199539789.013.0029

[B79] ^1^Rengier F. Hafner M. F. Unterhinninghofen R. Nawrotzki R. Kirsch J. Kauczor H. U. (2013). Integration of interactive three-dimensional image post-processing software into undergraduate radiology education effectively improves diagnostic skills and visual-spatial ability. *Eur. J. Radiol.* 82 1366–1371. 10.1016/j.ejrad.2013.01.010 23415424

[B80] ^1^Roesch A. Gruber H. Hawelka B. Hamm H. Arnold N. Popal H. (2003). Computer assisted learning in medicine: a long-term evaluation of the ‘Practical Training Programme Dermatology 2000’. *Med. Inform. Internet Med.* 28 147–159. 10.1080/14639230310001613430 14612304

[B81] ^1^Rubinstein J. Dhoble A. Ferenchick G. (2009). Puzzle based teaching versus traditional instruction in electrocardiogram interpretation for medical students - a pilot study. *BMC Med. Educ.* 9:4 10.1186/1472-6920-9-4 PMC2632655 19144134

[B82] Ruiz J. G. Mintzer M. J. Leipzig R. M. (2006). The impact of e-learning in medical education. *Acad. Med.* 81 207–212. 10.1097/00001888-200603000-00002 16501260

[B83] ^1^Salajegheh A. Jahangiri A. Dolan-Evans E. Pakneshan S. (2016). A combination of traditional learning and e-learning can be more effective on radiological interpretation skills in medical students: a pre- and post-intervention study. *BMC Med. Educ.* 16:46 10.1186/s12909-016-0569-5 PMC4739398 26842495

[B84] Salden R. J. C. M. Paas F. van Merriënboer J. J. G. (2006). Personalised adaptive task selection in air traffic control: effects on training efficiency and transfer. *Learn. Instr.* 16 350–362. 10.1016/j.learninstruc.2006.07.007

[B85] ^1^Schultz K. K. Brackbill M. L. (2009). Teaching electrocardiogram basics using dance and movement. *Am. J. Pharm. Educ.* 73:70 10.5688/aj730470 PMC2720366 19657503

[B86] ^1^Sendra-Portero F. Torales-Chaparro O. E. Ruiz-Gomez M. J. (2012). Medical students’ skills in image interpretation before and after training: a comparison between 3rd-year and 6th-year students from two different medical curricula. *Eur. J. Radiol.* 81 3931–3935. 10.1016/j.ejrad.2012.05.003 22647422

[B87] ^1^Sendra-Portero F. Torales-Chaparro O. E. Ruiz-Gomez M. J. Martinez-Morillo M. (2013). A pilot study to evaluate the use of virtual lectures for undergraduate radiology teaching. *Eur. J. Radiol.* 82 888–893. 10.1016/j.ejrad.2013.01.027 23434454

[B88] ^1^Shah R. Sibbald M. Jaffer N. Probyn L. Cavalcanti R. B. (2016). Online self-study of chest X-rays shows no difference between blocked and mixed practice. *Med. Educ.* 50 540–549. 10.1111/medu.12991 27072443

[B89] ^1^Silva C. S. Souza M. B. Silva R. S. de Medeiros L. M. Criado P. R. (2011). E-learning program for medical students in dermatology. *Clinics* 66 619–622. 10.1590/s1807-59322011000400016 21655756 PMC3093792

[B90] ^1^Singh D. G. Boudville N. Corderoy R. Ralston S. Tait C. P. (2011). Impact on the dermatology educational experience of medical students with the introduction of online teaching support modules to help address the reduction in clinical teaching. *Aust. J. Dermatol.* 52 264–269. 10.1111/j.1440-0960.2011.00804.x 22070700

[B91] ^1^Skinner A. A. Freeman R. V. Sheehan F. H. (2016). Quantitative feedback facilitates acquisition of skills in focused cardiac ultrasound. *Simul. Healthc.* 11 134–138. 10.1097/sih.0000000000000132 27043099

[B92] Spanjers I. A. Könings K. D. Leppink J. Verstegen D. M. de Jong N. Czabanowska K. (2015). The promised land of blended learning: quizzes as a moderator. *Educ. Res. Rev.* 15 59–74. 10.1016/j.edurev.2015.05.001

[B93] ^1^Tigges S. Lewis P. J. McNulty N. J. Mullins M. E. (2016). Medical student performance after a vertically integrated radiology clerkship. *J. Am. Coll. Radiol.* 13 67–71. 10.1016/j.jacr.2015.07.019 26499164

[B94] Van De Wiel M. W. Boshuizen H. P. Schmidt H. G. (2000). Knowledge restructuring in expertise development: evidence from pathophysiological representations of clinical cases by students and physicians. *Eur. J. Cogn. Psychol.* 12 323–356. 10.1080/09541440050114543

[B95] ^1^van den Berge K. van Gog T. Mamede S. Schmidt H. G. van Saase J. Rikers R. (2013). Acquisition of visual perceptual skills from worked examples: learning to interpret electrocardiograms (ECGs). *Interact. Learn. Environ.* 21 263–272. 10.1080/10494820.2011.554422

[B96] van der Gijp A. Ravesloot C. J. van der Schaaf M. F. van der Schaaf I. C. Huige J. C. Vincken K. L. (2015). Volumetric and two-dimensional image interpretation show different cognitive processes in learners. *Acad. Radiol.* 22 632–639. 10.1016/j.acra.2015.01.001 25704588

[B97] van der Gijp A. Schaaf M. F. Schaaf I. C. Huige J. C. B. M. Ravesloot C. J. Schaik J. P. J. (2014). Interpretation of radiological images: towards a framework of knowledge and skills. *Adv. Health Sci. Educ.* 19 565–580. 10.1007/s10459-013-9488-y 24449126

[B98] ^1^Van Es S. L. Kumar R. K. Pryor W. M. Salisbury E. L. Velan G. M. (2015). Cytopathology whole slide images and adaptive tutorials for postgraduate pathology trainees: a randomized crossover tirial. *Hum. Pathol.* 46 1297–1305. 10.1016/j.humpath.2015.05.009 26093936

[B99] ^1^Van Es S. L. Kumar R. K. Pryor W. M. Salisbury E. L. Velan G. M. (2016). Cytopathology whole slide images and adaptive tutorials for senior medical students: a randomized crossover trial. *Diagn. Pathol.* 11 1 10.1186/s13000-016-0452-z PMC4706725 26746436

[B100] Van Merrienboer J. J. G. Kirschner P. A. (2013). *Ten Steps to Complex Learning: A Systematic Approach to Four-Component Instructional Design*, 2nd Edn New York, NY: Routledge.

[B101] ^1^Varvaroussis D. P. Kalafati M. Pliatsika P. Castren M. Lott C. Xanthos T. (2014). Comparison of two teaching methods for cardiac arrhythmia interpretation among nursing students. *Resuscitation* 85 260–265. 10.1016/j.resuscitation.2013.09.023 24128798

[B102] Võ M. L. H. Aizenman A. M. Wolfe J. M. (2016). You think you know where you looked? You better look again. *J. Exp. Psychol. Hum. Percept. Perform.* 42 1477–1481. 10.1037/xhp0000264 27668308 PMC5079107

[B103] ^1^Webb A. L. Choi S. (2014). Interactive radiological anatomy eLearning solution for first year medical students: development, integration, and impact on learning. *Anatomical Sciences Education* 7 350–360. 10.1002/ase.1428 24376259

[B104] ^1^Williams J. Sato T. S. Policeni B. (2013). Pulmonary embolism teaching file: a simple pilot study for rapidly increasing pulmonary embolism recognition among new residents using interactive cross-sectional imaging. *Acad. Radiol.* 20 1048–1051. 10.1016/j.acra.2012.12.020 23506909

[B105] Zafar S. Safdar S. Zafar A. N. (2014). Evaluation of use of e-Learning in undergraduate radiology education: a review. *Eur. J. Radiol.* 83 2277–2287. 10.1016/j.ejrad.2014.08.017 25242658

[B106] ^1^Zeng R. Yue R. Z. Tan C. Y. Wang Q. Kuang P. Tian P. W. (2015). New ideas for teaching electrocardiogram interpretation and improving classroom teaching content. *Adv. Med. Educ. Pract.* 6 99–104. 10.2147/amep.s75316 25709515 PMC4329996

[B107] ^1^Zhang H. J. Hsu L. L. (2013). The effectiveness of an education program on nurses’ knowledge of electrocardiogram interpretation. *Int. Emerg. Nurs.* 21 247–251. 10.1016/j.ienj.2012.11.001 23266113

