# Supplementary material for: What We Do and Do Not Know about Teaching Medical Image Interpretation
Source: Front Psychol. 2017 Mar 3;8:309. doi: 10.3389/fpsyg.2017.00309 (PMC5334326; doi:10.3389/fpsyg.2017.00309)
Supplement: Supplementary file 1 [file Table_1.pdf]

What we Do and Do Not Know about Teaching Medical Image Perception

Ellen M Kok*, Koos van Geel, Jeroen J.G. van Merriënboer, Simon G. F. Robben

*** Correspondence:** Ellen Kok: e.kok@maastrichtuniversity.nl

Table 1. Overview of systematic search

	**reference**	**Topic**	**Findings**	**Domain**
1	(Ceresnak, Axelrod, Motonaga, Johnson, & Krawczeski, 2016)	whole curriculum/course	Pediatric cardiology residents enrolled in a pediatric cardiology "boot camp" had significantly higher post-test than pre-test scores, and evaluated the course positively.	ECG and radiology
2	(Cliff, Bedlow, Melia, Moss, & Harland, 2003)	whole curriculum/course	Medical students enrolled in a course involving a booklet on skin cancer plus a lecture had significantly higher post-test than pre-test scores.	Dermatology
3	(Darras et al., 2016)	Whole curriculum/course	Residents enrolled in a new radiology curriculum had significantly higher test scores than residents enrolled in the old curriculum, and were more positive about the curriculum.	Radiology
4	(Kohlwes & Shank, 2005)	whole curriculum/course	Residents had significantly higher post-test scores than pre-test scores after an ECG course.	ECG
5	(Lavranos, Koliaki, Briasoulis, Nikolaou, & Stefanadis, 2013)	whole curriculum/course	Medical students enrolled in a one-month ECG interpretation course had significantly higher post-test than pre-test scores.	ECG
6	(B. F. Lee, Chiu, & Li, 2013)	whole curriculum/course	Medical students enrolled in a case-based learning with traditional teaching curriculum evaluated the effectiveness of a nuclear medicine	Radiology

			curriculum more positively than students enrolled in a traditional teaching curriculum.	
7	(Liebman et al., 2012)	whole curriculum/course	Medical students enrolled in a dermoscopy tutorial improved significantly more in the diagnosis of cutaneous lesions compared to students who were not enrolled in the dermoscopy tutorial.	Dermatology
8	(Mahler, Wolcott, Swoboda, Wang, & Arnold, 2011)	whole curriculum/course	Medical students learned ECG interpretation through a workshop, a lecture or self-directed learning. All groups had significantly higher post-test than pre-test scores and scores were highest in the workshop and lecture groups.	ECG
9	(Ochsendorf, Boehncke, Boer, & Kaufmann, 2004)	whole curriculum/course	Students enrolled in a problem-oriented practical course and personal bedside teaching practical course had higher test-scores than students in a traditional course, and these courses were also more positively evaluated.	Dermatology
10	(Ochsendorf, Boehncke, Sommerlad, & Kaufmann, 2006)	whole curriculum/course	Students learned dermatology in a combination of large-group interactive teaching and small-group bedside teaching. This approach yielded higher scores and was more positively evaluated than a traditional course.	Dermatology
11	(Raupach, Brown, Anders, Hasenfuss, & Harendza, 2013)	whole curriculum/course	Students learned ECG interpretation in a self-directed learning course, lectures or a small-group peer-teaching course. No significant differences between test scores were found.	ECG
12	(Raupach et al., 2010)	whole curriculum/course	Students who learned ECG interpretation through near-peer teaching had a higher score than students in a lecture-based curriculum, but only when testing	ECG

This is a provisional file, not the final typeset article

			was formative, and not when it was summative.	
13	(Raupach, Harendza, Anders, Schuelper, & Brown, 2016)	whole curriculum/course	Students were enrolled in an ECG interpretation course through self-directed learning, lectures or small-group peer teaching. No differences between groups were found.	ECG
14	(Rengier et al., 2013)	whole curriculum/course	Medical students enrolled in a radiology course had significantly higher post-test than pre-test scores.	Radiology
15	(Sendra-Portero, Torales-Chaparro, & Ruiz-Gomez, 2012)	Whole curriculum/course	Medical students had significantly higher post-test scores after a radiology course than pre-test scores	Radiology
16	(Skinner, Freeman, & Sheehan, 2016)	whole curriculum/course	Residents improved their cardiac ultrasound acquisition and interpretation skills after a simulator-based curriculum.	Radiology
17	(Tigges, Lewis, McNulty, & Mullins, 2016)	whole curriculum/course	Students enrolled in a vertically integrated radiology clerkship had tests scores comparable to those of students at other medical schools.	Radiology
18	(Zeng et al., 2015)	whole curriculum/course	Students enrolled in a "graphics-sequence memory method" course had significantly higher test scores than students enrolled in a traditional course.	ECG
19	(Zhang & Hsu, 2013)	whole curriculum/course	Nurses enrolled in a lecture-based ECG course had significantly higher post-test than pre-test scores but test scores were not significantly different from nurses enrolled in a self-learning handbook course.	ECG
20	(Al-Rawi, Jacobs, Hassan,	e-learning	Oral health specialists, but not dental students, had significantly	Dental

	Sanderink, & Scarfe, 2007)		higher post-test than pre-test scores after interactive web-based education and all participant groups positively evaluated the course.	radiology
21	(Bailey, Roth, Kohli, & Heitkamp, 2014)	e-learning	Junior residents enrolled in an online training had significantly higher post-test than pre-test search pattern scores and also had significantly higher test scores than untrained residents.	Radiology
22	(Boespflug, Guerra, Dalle, & Thomas, 2015)	e-learning	Dermatologists/dermatology residents enrolled in an e-learning course had significantly higher test scores than participants of the control group and the e-learning course was positively evaluated.	Dermatology
23	(Bojsen et al., 2015)	e-learning	Medical students enrolled in a web-based ECG interpretation training had significantly higher scores on an immediate post-test than the pre-test but not on retention tests.	ECG
24	(Breen, Zhu, Bond, Finlay, & Clifford, 2016)	e-learning	Students enrolled in an online e-learning training had non-significantly higher post-test than pre-test scores and positively evaluated the course.	ECG
25	(Burbridge, Kalra, Malin, Trinder, & Pinelle, 2015)	e-learning	Medical students enrolled in an e-learning course had significantly higher test scores than students in the control group and they evaluated the course positively.	Radiology
26	(Chudakoff, Obuchowski, Mehta, & Reid, 2013)	e-learning	Participants using a web-based pediatric radiology curriculum improved on average by 16.45% and evaluated the curriculum positively.	Radiology
27	(Chudgar, Engle, Grochowski, &	e-learning	Medical students enrolled in a self-directed e-learning ECG course had significantly higher post-test	ECG

This is a provisional file, not the final typeset article

	Gagliardi, 2016)		than pre-test scores and also had significantly higher test-scores than a previous group, which did not have access to the course.	
28	(DeBonis, Blair, Payne, Wigan, & Kim, 2015)	e-learning	Psychiatry residents enrolled in an e-learning ECG-reading course had non-significant higher post-test than pre-test scores and evaluated the training positively.	ECG
29	(Fawcett, Widmaier, & Cavanaugh, 2004)	e-learning	Family medicine residents who actively participated in a digital dermatology instructional program scored significantly higher on post-tests than a pre-test than residents who did not actively participate, but over time the difference in test scores diminished.	Dermatology
30	(Giunta, Di Stefani, & Chimenti, 2011)	e-learning	Medical students enrolled in an e-learning dermatology course had significantly higher test scores than students without access to this course.	Dermatology
31	(Jenkins, Goel, & Morrell, 2008)	e-learning	Medical students enrolled in an e-learning dermatology course had non-inferior test scores compared to students who followed a lecture.	Dermatology
32	(Kavadella, Tsiklakis, Vougiouklakis, & Lionarakis, 2012)	e-learning	Students enrolled in a blended learning course on dental radiology had significantly higher test scores than students enrolled in a traditional course and students evaluated the blended learning course positively.	Dental radiology
33	(Ketelsen et al., 2007)	e-learning	Students enrolled in an e-learning course had non-inferior test scores compared to students who had a lecture course or printed text course; students preferred a mixture between lectures and e-learning.	Radiology

34	(Lieberman, Abramson, Volkan, & McArdle, 2002)	e-learning	Students enrolled in an interactive e-learning course scored non-inferior to students enrolled in an interactive tutorial course.	Radiology
35	(Lim-Dunham, Ensminger, McNulty, Hoyt, & Chandrasekhar, 2016)	e-learning	The mean radiology score for students for whom an online radiology curriculum was mandatory was significantly higher than for students for whom the curriculum was voluntary.	Radiology
36	(Mahnken, Baumann, Meister, Schmitt, & Fischer, 2011)	e-learning	Medical students enrolled in an elective or mandatory e-learning course scored non-significantly higher on a post-test than students without access to the e-learning course.	Radiology
37	(Maleck et al., 2001)	e-learning	Students enrolled in a paper-based or e-learning course had significantly higher post-test than pre-test scores, in contrast to students without exposure to any course.	Radiology
38	(Maloney, Hippe, Paladin, Chew, & Ha, 2016)	e-learning	Radiology residents enrolled in an e-learning course scored significantly higher on the post-test than pre-test but scores were not significantly different from scores of radiology residents who followed a lecture-based course.	Radiology
39	(Meckfessel et al., 2011)	e-learning	Students positively evaluated an e-learning course on dental radiology and failure rates on the final exam were lower than the failure rates of previous years, when the e-learning course was not yet implemented.	Dental radiology
40	(Mileman, van den Hout, & Sanderink, 2003)	e-learning	Students with access to a computer-assisted learning program before the test scored significantly higher than a group with no access to the	Dental radiology

This is a provisional file, not the final typeset article

			program before the test.	
41	(Montassier et al., 2016)	e-learning	Medical students enrolled in an e-learning course on ECG reading scored significantly higher on the post-test than pre-test but scored not significantly different from students enrolled in a lecture-based course.	ECG
42	(Nilsson et al., 2008)	e-learning	Medical students enrolled in an e-learning ECG course had significantly higher test scores than students who did not have access to the course.	ECG
43	(O'Connor et al., 2016)	e-learning	Medical students enrolled in an e-learning course had significantly higher post-test than pre-test scores, but scores were not significantly different from students enrolled in a traditional course.	Radiology
44	(Pagnanelli et al., 2003)	e-learning	Dermatologists enrolled in an e-learning course on dermatoscopy had significantly higher post-test than pre-test scores.	Dermatology
45	(Porras et al., 2016)	e-learning	Sports medicine fellows enrolled in an e-learning course on ECG interpretation had significantly higher post-test than pre-test scores.	ECG
46	(Porter, Bailey, Woods, Scott, & Johnson, 2015)	e-learning	Radiology residents enrolled in an e-learning course had significantly higher post-test than pre-test scores and felt more confident in their interpretation skills.	Radiology
47	(Roesch et al., 2003)	e-learning	Medical students enrolled in an e-learning course scored higher on a test than students who were not enrolled and evaluated the e-	Dermatology

			learning course positively.	
48	(Salajegheh, Jahangiri, Dolan-Evans, & Pakneshan, 2016)	e-learning	Medical students enrolled in an e-learning course had significantly higher post-test scores than students who were not enrolled, but no significant retention of knowledge after one year was found.	Radiology
49	(Sendra-Portero, Torales-Chaparro, Ruiz-Gomez, & Martinez-Morillo, 2013)	e-learning	Medical students enrolled in an e-learning course had significantly higher test scores than students enrolled in a traditional course and students evaluated the e-learning course positively.	Radiology
50	(Silva, Souza, Silva, de Medeiros, & Criado, 2011)	e-learning	Students enrolled in an e-learning course had significantly higher post-test than pre-test scores which were also significantly higher than the post-test scores of students who did not have access to the e-learning course.	Dermatology
51	(Singh, Boudville, Corderoy, Ralston, & Tait, 2011)	e-learning	Students enrolled in an e-learning course self-assessed their knowledge and skills to be higher than students of cohorts who did not have access to an e-learning course.	Dermatology
52	(Van Es, Kumar, Pryor, Salisbury, & Velan, 2015)	e-learning	Students enrolled in an e-learning course did not have significantly higher test scores than students enrolled in a traditional course, but the e-learning course was positively evaluated.	Pathology
53	(Van Es, Kumar, Pryor, Salisbury, & Velan, 2016)	e-learning	Medical students enrolled in an e-learning course did not have significantly higher test scores than students enrolled in a traditional course, but the e-learning course was positively evaluated.	Pathology

This is a provisional file, not the final typeset article

54	(Webb & Choi, 2014)	e-learning	Students enrolled in an e-learning course had significantly higher post-test than pre-test scores and evaluated the e-learning course positively.	Radiology
55a	(Williams, Sato, & Policeni, 2013)	e-learning	Radiology residents enrolled in a radiology e-learning course had significantly higher post-test than pre-test scores.	Radiology
55b	(Ark, Brooks, & Eva, 2006)	specific interventions (diagnostic reasoning)	Students who learned ECG interpretation through a combined reasoning strategy (be feature-oriented and trust similarity) improved performance more than students who were instructed to be only feature-oriented or only trust similarity.	ECG
56	(Auffermann, Henry, Little, Tigges, & Tridandapani, 2015)	specific interventions (diagnostic reasoning)	Students enrolled in a nodule detection course at simulated work stations identified significantly more nodules on a test and positively evaluated the course.	Radiology
57	(Auffermann, Little, & Tridandapani, 2016)	specific interventions (diagnostic reasoning)	Students trained to use a search pattern to detect nodules in radiographs improved significantly more than students who did not receive training.	Radiology
58	(Baghdady, Carnahan, Lam, & Woods, 2014a)	specific interventions (diagnostic reasoning, schemas)	Students learning dental radiology by providing a diagnosis first, and subsequently listing features had a higher diagnostic accuracy than students who listed features first, and subsequently provided a diagnosis.	Dental radiology
59	(Baghdady, Pharoah, Regehr, Lam, & Woods, 2009)	specific interventions (schemas)	Students who learned diagnostic oral radiology by studying a basic-science elaboration scored significantly higher on a post-test than students who studied a	Dental radiology

			structured algorithm or feature list.	
60	(Baghdady, Carnahan, Lam, & Woods, 2014b)	specific interventions (Study strategies)	Students in a test-enhanced condition had a higher test score than students in a study condition.	Dental radiology
61	(Blissett, Cavalcanti, & Sibbald, 2015)<br><br>experiment 1	specific interventions (schemas)	Students enrolled in a course with an expert-generated ECG interpretation schema had better test scores than students in a traditional course.	ECG
62	(Blissett et al., 2015)<br><br>experiment 2	specific interventions (schemas)	Students studying an expert-generated schema learned ECG interpretation better than students who studied learner-generated schemas.	ECG
63	(Dong et al., 2015)	specific interventions (schemas)	Students studying ECG interpretation by making concept maps had significantly higher test scores than students taught a traditional teaching format.	ECG
64	(Eva, Hatala, LeBlanc, & Brooks, 2007)	specific interventions (diagnostic reasoning)	Students scored significantly higher on ECG interpretation when instructed to combine non-analytical reasoning and analytical reasoning as compared to a group without reasoning instruction.	ECG
65	(Garg, Haley, & Hatem, 2010)	specific interventions (schemas)	Students who learned dermatology through studying 3D prosthetic mimics of lesions had significantly higher test scores than students who studied 2D images.	Dermatology
66	(Hatala, Brooks, & Norman, 2003)	specific interventions (schemas)	Medical students enrolled in a mixed practice course scored significantly higher than students enrolled in a blocked practice course.	ECG
67	(Hecht, Adams, Cunningham, Lane, & Howell,	specific interventions (other)	After the introduction of clickers during radiology lectures, medical students were significantly more	Radiology

This is a provisional file, not the final typeset article

	2013)		positive towards lectures but exam scores did not significantly differ.	
68	(Kaliyadan, Amri, Dhufiri, Amin, & Khan, 2012)	specific interventions (other)	Students enrolled in a tutorless problem-based learning course did not have significantly different test scores from students enrolled in traditional problem-based learning.	Dermatology
69	(Kok, de Bruin, Robben, & van Merrienboer, 2013)	specific interventions (schemas)	Medical students who were enrolled in comparison learning training with normal images identified significantly more focal diseases on the post-test than students who compared with the same diseases.	Radiology
70	(Kok, de Bruin, Leppink, van Merrienboer, & Robben, 2015)	specific interventions (schemas)	Medical students enrolled in comparison learning with comparisons of different-diseases and comparisons of same-diseases were most efficient in learning radiology compared to students enrolled in other types of comparison learning or a control condition.	Radiology
71	(Kok et al., 2016)	specific interventions (diagnostic reasoning)	Medical students enrolled in systematic-viewing training did not find significantly more abnormalities on the post-test than students enrolled in full-coverage training or non-systematic viewing training.	Radiology
72	(H. Lee et al., 2010)	specific interventions (schemas)	Significantly more veterinary students could deduce pathophysiological principles underlying radiological changes after education with 3D CT volume rendering models than students who received conventional x-ray education.	Radiology
73	(Li et al., 2013)	specific interventions (other)	Medical students enrolled in one of three problem-based courses (real-patient cases, digital cases or paper	Dermatology

			cases) on dermatology had better results than students in a lecture-based course. Overall results were highest for the group with real-patient cases.	
74	(Marsch, Espiritu, Groth, & Hutchens, 2014)	specific interventions (other)	Medical students enrolled in a pathology training in which abnormalities were annotated scored significantly higher on the post-test than students enrolled in a pathology training in which abnormalities were not annotated.	Pathology
75	(Pusic et al., 2012)	specific interventions (schemas)	Residents enrolled in a training with a high proportion of abnormal images identified significantly more abnormalities on a post-test, while residents enrolled in a training with a low proportion of abnormal images identified significantly more normal images on the post-test.	Radiology
76	(Pusic, LeBlanc, & Miller, 2007)	specific interventions (other)	Medical students enrolled in a training with a fixed order lay-out did not have significantly different test scores from students enrolled in a training with a branched order lay-out.	Radiology
77	(Rubinstein, Dhoble, & Ferenchick, 2009)	specific interventions (other)	Medical students enrolled in a puzzle-based ECG course had significantly higher post-test than pre-test scores, but scores were not significantly different from post-test scores of students enrolled in a traditional course.	ECG
78	(Schultz & Brackbill, 2009)	specific interventions (other)	Medical students enrolled in a dance-based ECG course had significantly higher post-test than pre-test scores, but the scores were not significantly different from post-test scores of students enrolled in a traditional course.	ECG

This is a provisional file, not the final typeset article

79	(Shah, Sibbald, Jaffer, Probyn, & Cavalcanti, 2016)	specific interventions (study strategies)	Medical students enrolled in a blocked-practice radiology course did not have significantly different test scores compared to medical students enrolled in a mixed-practice course.	Radiology
80	(Varvaroussis et al., 2014)	specific interventions (diagnostic reasoning)	Nursing students enrolled in a systematic-viewing course on ECG interpretation did not have significantly higher post-test scores than students enrolled in a non-systematic viewing course.	ECG
81	(van den Berge et al., 2013)	specific interventions (schemas)	Participants who studied ECG worked examples had higher post-test scores than participants who had to describe the ECGs themselves.	ECG
